# The Future Life

**Published:** 1889-10

**Authors:** Victor Hugo


### THE FUTURE LIFE.

I feel in myself the future life. I am like a forest which has been more than once cut down. The new shoots are stronger and livelier than ever. I am rising, I know, toward the sky. The sunshine is over my head. The earth gives me its generous sap, but heaven lights me with the reflection of unknown worlds.

You say the soul is nothing but the resultant of bodily powers; why then is my soul the more luminous when my bodily powers begin to fail? Winter is on my head and eternal spring is in my heart. Then I breathe at this hour, the fragrance of the lilies, the violets, and the roses as at twenty years. The nearer I approach the end, the plainer I hear around me the immortal symphonies of the worlds which unite me. It is marvelous, yet simple. It is a fairy tale, and it is history.

For half a century I have been writing my thoughts in prose, verse, history, philosophy, drama, romance, tradition, satire, ode, song—I have tried all. But I feel that I have not said the thousandth part of what is in me. When I go down to the grave I can say, like so many others, "I have finished my day's work;" but I cannot say, "I have finished my life." My day's work will begin again the next morning. The tomb is not a blind alley, it is a thoroughfare. It closes in the twilight to open with the dawn.

I improve every hour because I love this world as my fatherland. My work is only a beginning. My work is hardly above its foundation. I would be glad to see it mounting and mounting forever. The thirst for the infinite proves infinity.—*Victor Hugo.*

